# Supplementary material for: Zebrafish Xenografts Unveil Sensitivity to Olaparib beyond BRCA Status
Source: Cancers (Basel). 2020 Jul 2;12(7):1769. doi: 10.3390/cancers12071769 (PMC7408583; doi:10.3390/cancers12071769)
Supplement: Supplementary file 1 [file cancers-12-01769-s001.pdf]

Supplementary Material: Zebrafish Xenografts Unveil Sensitivity to Olaparib beyond BRCA Status

Ana Beatriz Varanda, Ana Martins-Logrado, Miguel Godinho Ferreira and Rita Fior

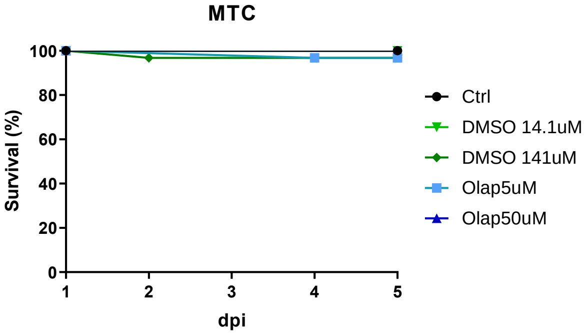

Figure S1. Concentration of dimethyl sulfoxide (DMSO) and Olaparib in zebrafish larvae medium (E3). Zebrafish survival at different concentrations of DMSO and Olaparib in the fish water (E3). DMSO alone was diluted in E3 at the indicated concentrations. Olaparib was dissolved in DMSO and stored at −20 °C. Upon usage, the stock solution was further diluted in E3 to the indicated concentrations. *n* = 100 larvae in each condition.

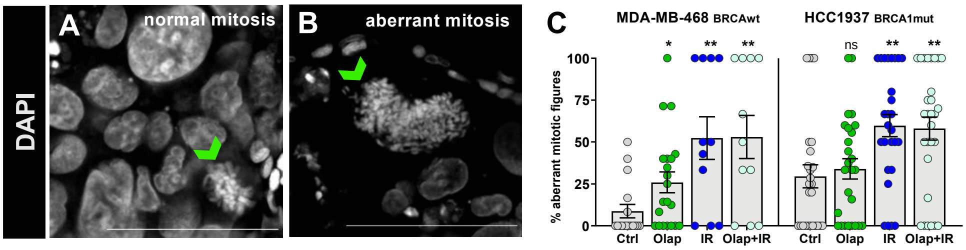

Figure S2. Olaparib and IR can lead to aberrant mitosis. TNBC cell lines (MDA-MB-469^{BRCAwt} and Hcc1937^{BRCA1mut}) were fluorescently labeled with CM-DiI (not shown*)* and injected in the perivitelline space (PVS) of 2dpf zebrafish larvae. Zebrafish xenografts were screened and randomly distributed amongst experimental conditions; treatment regimens were started at 24 h post injection (24hpi). Zebrafish xenografts were sacrificed and fixed at 5dpi and imaged by confocal microscopy with DAPI staining (in grey). (**A**,**B**). The green arrowheads illustrate examples of the quantified mitotic figures. The quantification of aberrant mitotic figures was normalized to their respective controls (**C**)**.** Results are from 3 independent experiments and expressed as mean ± SEM, each dot represents one xenograft. The attribution of an aberrant status was based on the identification of mitotic abnormalities such as chromatin bridges, lagging chromosomes or asymmetric mitosis. Statistical results: not significant (ns) > 0.05, * $p \leq 0.05$, ** $p \leq 0.01$.

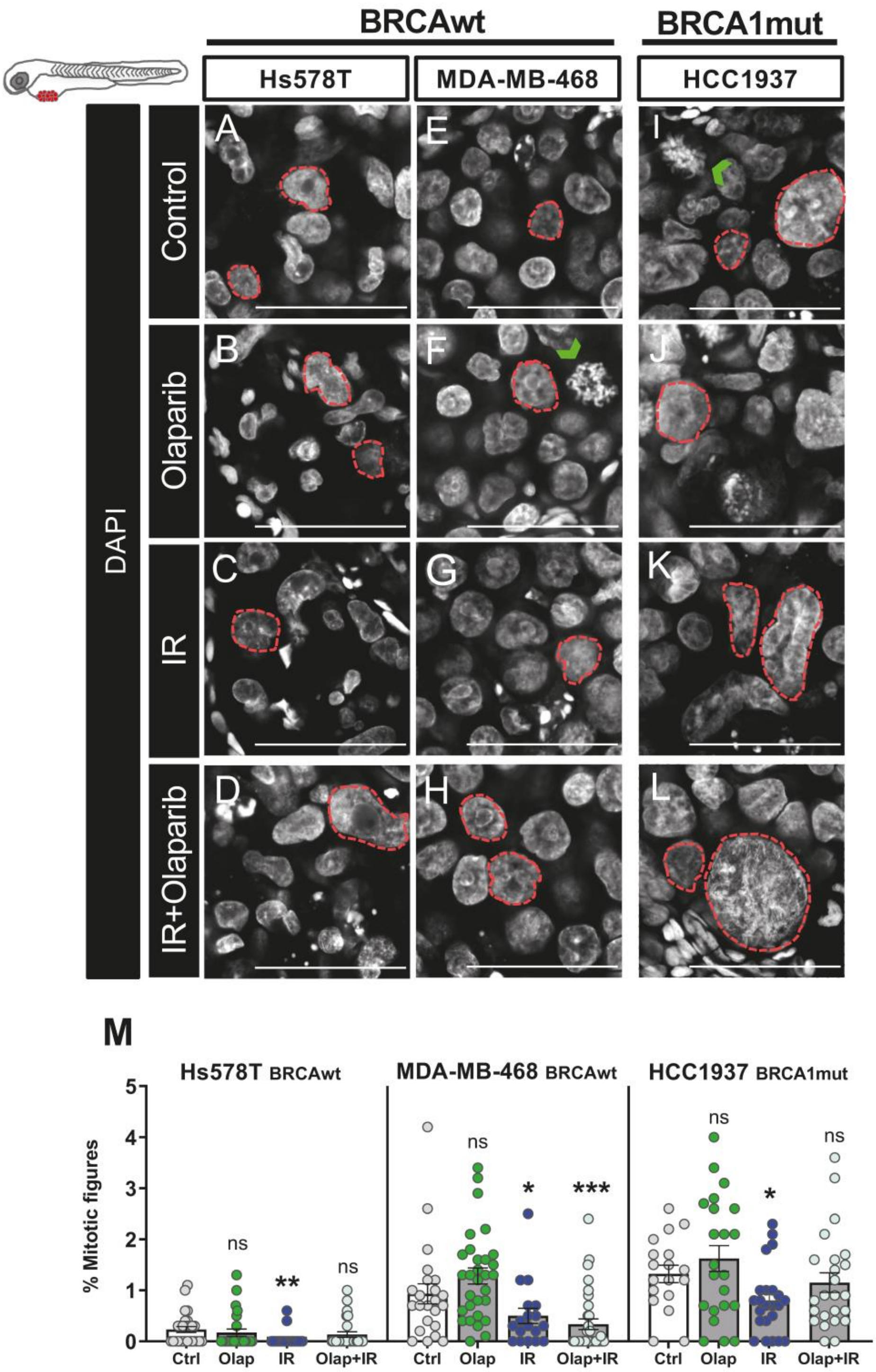

Figure S3. Single-cell resolution nuclear morphology changes upon challenge with Olaparib and/or IR. TNBC cell lines (Hs578T^{BRCAwt}, MDA-MB-469^{BRCAwt} and Hcc1937^{BRCA1mut}) were fluorescently labeled with CM-DiI (not shown*)* and injected in the perivitelline space (PVS) of 2dpf zebrafish larvae. Zebrafish xenografts were screened and randomly distributed amongst experimental conditions; treatment regimens were started at 24 h post injection (24hpi). Zebrafish xenografts were sacrificed and fixed at 5 days post injection (dpi) and imaged by confocal microscopy with DAPI staining (in grey). (**A–L**). The green arrowheads illustrate examples of the quantified mitotic figures. The quantification of mitotic figures is represented in percentage in relation to total number of cells. (**M**)**.** Results are from 3 independent experiments and expressed as mean ± SEM, each dot represents one xenograft. Statistical results: not significant (ns) > 0.05, * $p \leq 0.05$, ** $p \leq 0.01$, *** $p \leq 0.001$. The dashed line represents tumor nuclear area. Scale bar: 50 µm.

Table S1. Characteristics of the TNBC cell lines used, origin, molecular classification, DDR status, mutations and known response to olaparib.

Characterization	Hs578T	MDA-MB-468	HCC1937	Ref.
Origin/Disease	Human mammary gland/Carcinoma	Human mammary gland; derived from metastatic site: pleural effusion/Adenocarcinoma	Human mammary gland/Primary ductal carcinoma	[1]
Molecular classification	Basal B/MSL	Basal A/BL1	Basal A/BL1	[2]
BRCA status	Wt<br>BRCA1 allelic loss	wt<br>BRCA1 allelic loss	Mut—BRCA1 5382insC<br>BRCA1 allelic loss	[1,3]
HRR status	Proficient	Controversial	Controversial	[4–6]
TP53	Mut	Mut	Mut	[1]
PI3K pathway	wt	PTEN homo deletion	PTEN homo deletion	[1]
Rb	wt	Mut	Mut	[7]
Response to olaparib	Resistant	Sensitive	Moderate sensitivity	[2,5,8–10]
Response to IR	Moderate Sensitivity	Sensitive	Sensitive	[6,7,9,11]

References

1. Chavez, K.J.; Garimella, S.V.; Lipkowitz, S. Triple Negative Breast Cancer Cell Lines: One Tool in the Search for Better Treatment of Triple Negative Breast Cancer. *Breast Dis.* **2012**, *32*, 35–48.
2. Lehmann, B.D.; Bauer, J.A.; Chen, X.; Sanders, M.E.; Chakravarthy, A.B.; Shyr, Y.; Pietenpol, J.A. Identification of human triple-negative breast cancer subtypes and preclinical models for selection of targeted therapies. *J. Clin. Investig.* **2011**, *121*, 2750–2767.
3. Elstrodt, F.; Hollestelle, A.; Nagel, J.H.A.; Gorin, M.; Wasielewski, M.; Van Den Ouweland, A.; Merajver, S.D.; Ethier, S.P.; Schutte, M. BRCA1 Mutation Analysis of 41 Human Breast Cancer Cell Lines Reveals Three New Deleterious Mutants. *Cancer Res* **2006**, *66*, 41–46.
4. Popova, T.; Manié, E.; Rieunier, G.; Caux-Moncoutier, V.; Tirapo, C.; Dubois, T.; Delattre, O.; Sigal-Zafrani, B.; Bollet, M.; Longy, M.; et al. Ploidy and large-scale genomic instability consistently identify basal-like breast carcinomas with BRCA1/2 inactivation. *Cancer Res.* **2012**, *72*, 5454–5462.
5. Peng, G.; Lin, C.C.J.; Mo, W.; Dai, H.; Park, Y.Y.; Kim, S.M.; Peng, Y.; Mo, Q.; Siwko, S.; Hu, R.; et al. Genome-wide transcriptome profiling of homologous recombination DNA repair. *Nat. Commun.* **2014**, *5*, 1–11.
6. Hill, S.J.; Clark, A.P.; Silver, D.P.; Livingston, D.M. BRCA1 Pathway Function in Basal-Like Breast Cancer Cells. *Mol. Cell. Biol.* **2014**, *34*, 3828–3842.
7. Robinson, T.J.W.; Liu, J.C.; Vizeacoumar, F.; Sun, T.; Maclean, N.; Egan, S.E.; Schimmer, A.D.; Datti, A.; Zacksenhaus, E. RB1 status in triple negative breast cancer cells dictates response to radiation treatment and selective therapeutic drugs. *PLoS ONE* **2013**, *8*, e78641.
8. Keung, M.Y.; Wu, Y.; Badar, F.; Vadgama, J.V. Response of Breast Cancer Cells to PARP Inhibitors Is Independent of BRCA Status. *J. Clin. Med.* **2020**, *9*, 940.
9. Lafontaine, J.; Boisvert, J.S.; Glory, A.; Coulombe, S.; Wong, P. Synergy between non-thermal plasma with radiation therapy and olaparib in a panel of breast cancer cell lines. *Cancers (Basel)* **2020**, *12*, 348.
10. Yang, K.S.; Kohler, R.H.; Landon, M.; Giedt, R.; Weissleder, R. Single cell resolution in vivo imaging of DNA damage following PARP inhibition. *Sci. Rep.* **2015**, *5*, 10129.
11. Ree, A.H.; Bratland, Å.; Landsverk, K.S.; Fodstad, Ø. Ionizing Radiation Inhibits the PLK Cell Cycle Gene in a G2 Checkpoint-dependent Manner. *Anticancer Res.* **2004**, *24*, 555–562.

© 2020 by the authors. Licensee MDPI, Basel, Switzerland. This article is an open access article distributed under the terms and conditions of the Creative Commons Attribution (CC BY) license (http://creativecommons.org/licenses/by/4.0/).
